# Analysis of factors related to preterm birth: a retrospective study at Nanjing Maternity and Child Health Care Hospital in China

**DOI:** 10.1097/MD.0000000000021172

**Published:** 2020-07-10

**Authors:** Jin Huang, Yating Qian, Mingming Gao, Hongjuan Ding, Lei Zhang, Ruizhe Jia

**Affiliations:** a Department of Obstetrics and Gynecology, Obstetrics and Gynecology Hospital Affiliated to Nanjing Medical University, Nanjing, Jiangsu 210004; b Nanjing Maternity and Child Health Care Hospital, Women's Hospital of Nanjing Medical University, Nanjing, Jiangsu 210004; c Yixing People's Hospital, Jiangsu 214200, P.R. China.

**Keywords:** heterogeneity, interaction, multivariate, preterm birth

## Abstract

Preterm birth is the most important cause of neonatal mortality and morbidity worldwide. The aim of this study was to identify factors associated with preterm birth and examine the heterogeneity and interactions between these factors.

We collected data from 1607 pregnant women treated at Nanjing Maternity and Child Health Care Hospital in China. The women included in the study were divided into the full-term group and the preterm-birth group. We used *t*-tests to compare the characteristics of age and body mass index, Chi-square tests for the other variables, and we used the Wald test to calculate the interaction between factors that may affect preterm birth. The heterogeneity test was used to study the relationship between subgroups. Multivariable logistic regression analysis was used to explore the associations between risk factors and preterm birth, which included all risk factors. All tests were 2-tailed, *P* < 0.05 was considered significant, and 95% confidence intervals were estimated for percentages.

There was no statistical difference in basic characteristics such as age between the full-term and preterm groups. We found 6 independent risk factors that were associated with preterm birth (*P* < .05): preeclampsia (PE), intrahepatic cholestasis, premature rupture of the membranes (PROM), placenta previa, chorioamnionitis, and scarred uterus. Five combinations of these factors were statistically significant (*P* < .05) in terms of heterogeneity: PE and PROM; placenta previa and polyhydramnios; chorioamnionitis and PE; PROM and maternal body mass index; and PROM and gestational diabetes mellitus. Ultimately, the 2 subgroups that showed interactions were PE and PROM and chorioamnionitis and PE.

The interaction between different factors over the course of preterm birth cannot be ignored. When independent risk factors are combined with other diseases, such as PE combined with PROM or chorioamnionitis in this study, it may more likely result in preterm birth. Thus, this situation deserves particular clinical attention.

## 1 Introduction

Preterm birth, an important cause of perinatal morbidity and mortality, is defined as delivery before 37 weeks (259 days) of gestation, according to the guidelines of the World Health Organization. As a serious social and health problem, the rate of preterm birth is 5% to 13% in most countries, resulting in 15 million preterm deliveries worldwide each year. Premature babies have an increased risk of death, and compared with term infants, premature babies are more likely to develop long-term neurological and developmental disorders.^[1,2]^ In addition, preterm birth can also increase the risk of death from other neonatal diseases.^[3]^

Preterm birth is a highly complex process, influenced by multiple factors. According to recent studies, lifestyle and physiological conditions of the mother, such as maternal weight and smoking, are high-risk factors for preterm birth.^[2]^ For example, 1 report showed that smokers have significantly higher rates of preterm birth than nonsmokers, and that quitting smoking early in pregnancy can reduce adverse pregnancy outcomes.^[4]^ The obstetric causes of preterm birth are mainly divided into medical indications (including maternal and fetal indications), premature rupture of the membranes (PROM), and spontaneous preterm birth. Approximately 30% to 35% of all preterm births are caused by medical indications, 40% to 45% are caused by spontaneous preterm birth, and 25% to 30% are caused by PROM.^[5]^

The aim of this comprehensive study was to identify factors associated with singleton preterm birth and to determine whether the superposition of factors impacts preterm birth, to enable a greater focus on these conditions and to attempt to reduce the incidence of preterm births.

## 2 Materials and methods

### 2.1 Study population

**Figure 1:**
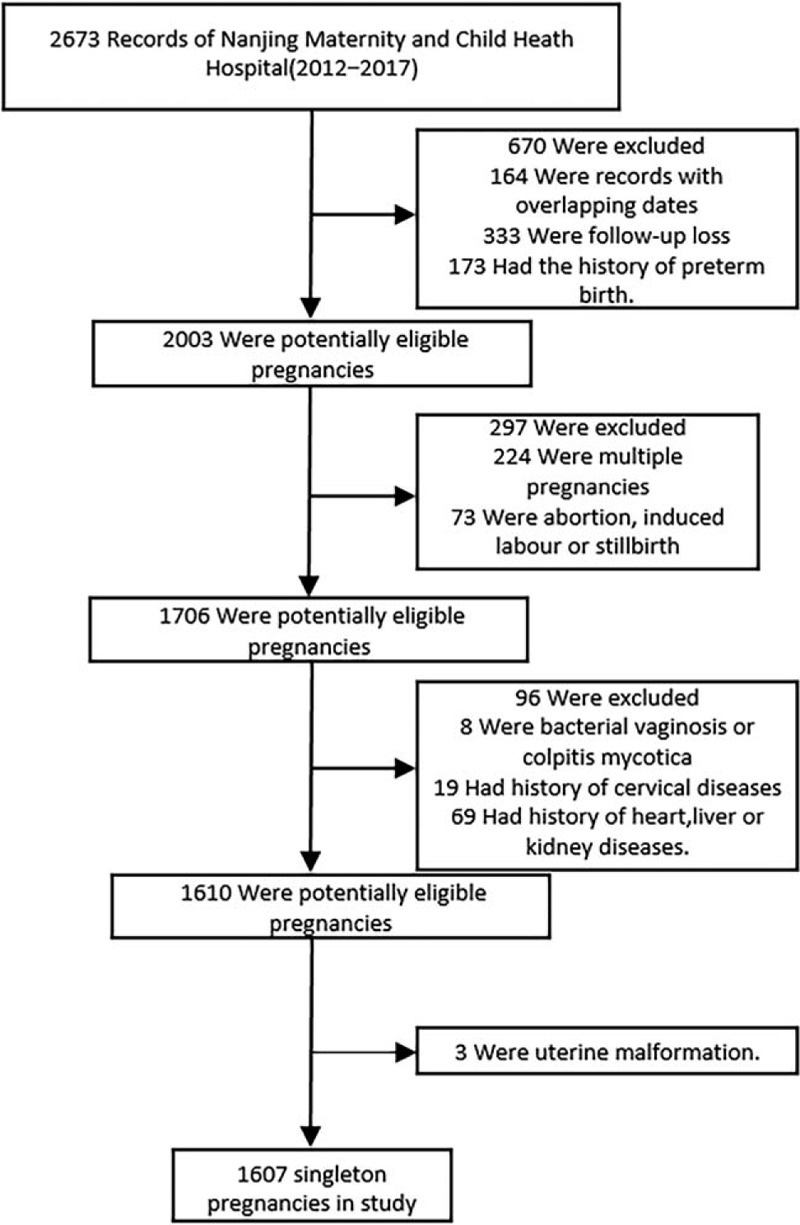
Flow of participants in the study.

We performed a retrospective study and collected data by the random number method regarding 2673 pregnant women admitted to Nanjing Maternity and Child Health Care Hospital in China from 2012 to 2017. Of these women, we sequentially excluded women for whom the birth records were either lost (n = 333) or duplicated (n = 164) during follow-up. Women with a history of preterm birth (n = 173) were also excluded. Next, we excluded women who had multiparous pregnancies (n = 224) or abortions, induced labor, or stillbirths (n = 73). We also excluded women who had bacterial vaginosis or colpitis mycotica (n = 8) or cervical diseases (n = 19) during pregnancy, and women who had a history of heart, liver, or kidney diseases (n = 69). Finally, women with uterine malformations (n = 3) were also excluded. A total of 1607 singleton pregnancies were included for analysis (Fig. 1). All women included in this study were nonsmokers.

### 2.2 Ethics

Written consent was obtained from all women, and the study was approved by the Research Ethics Committee of Nanjing Medical University as well as the Nanjing Maternal and Child Health Hospital and the Obstetrics and Gynecology Hospital Affiliated with Nanjing Medical University.

### 2.3 Statistical analysis

We used Chi-square tests for categorical variables and *t*-tests for continuous variables to compare the characteristics of the women in this study. Continuous variables are expressed as the mean ± standard deviation, while categorical variables are presented as frequencies and percentages. In this study, there was normal distribution of both age and body mass index (BMI). We counted all instances of the diseases affecting the population under study, and these were included in the test. Multivariable logistic regression analysis was used to explore the association between risk factors and preterm birth, which included all risk factors in Table 1 as predictors to adjust for confounding variables. Crude and adjusted odds ratios (OR) with 95% confidence intervals (95% CI) are presented. These statistical analyses were conducted using the statistical software package R 3.5.0. We divided participants into different subgroups using Stata 15.1 software to calculate the *P*-value for heterogeneity between subgroups. We hypothesized that interactions could exist between the independent risk factors and preterm birth; therefore, these interaction terms were also assessed in our study using the Wald test. All tests were 2-tailed, *P* < .05 was considered significant, and 95% CI values were estimated for the percentages.

**Table 1:**
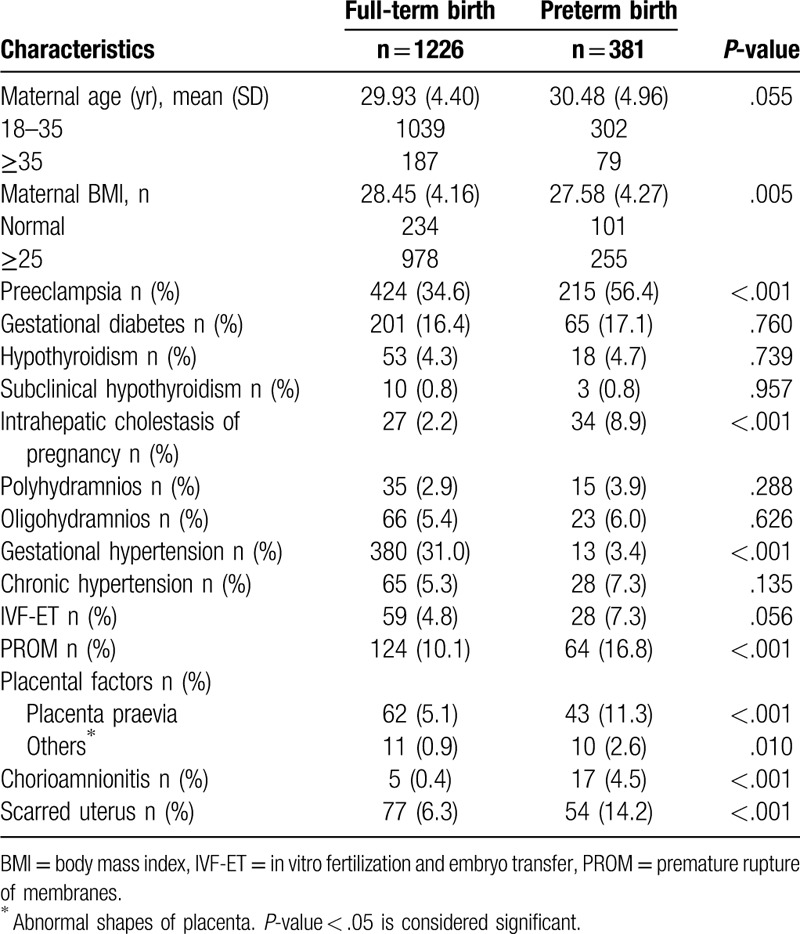
Comparison between women with term and preterm birth.

## 3 Results

The study population consisted of 1607 women. Demographic and clinical characteristics for full-term and preterm cases are shown in Table 1. Preterm cases represented approximately 23.7% (381/1607) of our total population. Women with preterm births (n = 381) were compared with women who had full-term births (n = 1226). There was no significant difference in maternal age between the 2 groups (*P* = 0.055). As shown in Table 1, those who had preeclampsia (PE), intrahepatic cholestasis of pregnancy (ICP), PROM, placenta previa, or chorioamnionitis were more likely to experience preterm births. The proportion of women with a scarred uterus in preterm birth was higher than that in full-term birth (14.2% vs 6.3%, *P* < .001, Chi-square test). The BMI and other placental factors (such as battledore placenta) seemed to have an impact on preterm birth (BMI: *P* = .005; other placental factors: *P* = .010).

Interestingly, among the entire group of 1,607 women, more women with gestational hypertension underwent full-term births (31%) than preterm births (3.4%). We considered that gestational hypertension may be transient, or it may represent early (before proteinuria) PE.^[6]^ Additionally, the conditions of these women may be milder, and their time of onset may be later than that of individuals who experienced preterm birth.

### 3.1 Multivariable logistic regression

**Figure 2:**
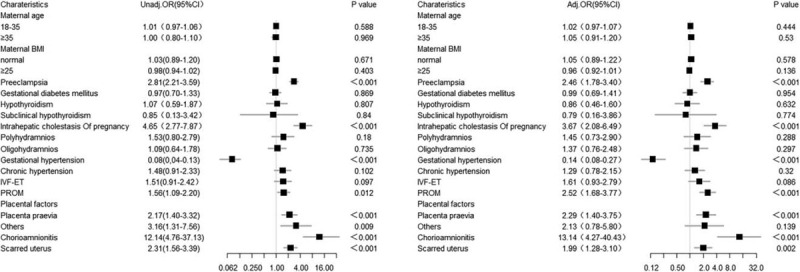
Unadjusted and adjusted odds ratios of clinical characteristics and other diseases in Table 1 for preterm birth, using multivariable logistic regression. *P* < .05 was considered significant.

Because preterm birth is likely to be the result of multiple factors,^[7]^ we carried out multivariable logistic regression analyses on the factors in Table 1 to explore independent risk factors for preterm birth. The results are shown in Figure 2. Following adjustment for all the variables listed in Table 1, we determined that 6 independent risk factors were associated with preterm birth: PE (unadjusted OR 2.81, 95% CI 2.21–3.59 and adjusted OR 2.46, 95% CI 1.78–3.40), ICP (unadjusted OR 4.65, 95% CI 2.77–7.87 and adjusted OR 3.67, 95% CI 2.08–6.49), PROM (unadjusted OR 1.56, 95% CI 1.09–2.20 and adjusted OR 2.52, 95% CI 1.68–3.77), placenta previa (unadjusted OR 2.17, 95% CI 1.40–3.32 and adjusted OR 2.29, 95% CI 1.40–3.75), chorioamnionitis (unadjusted OR 12.14, 95% CI 4.76–37.13 and adjusted OR 13.14, 95% CI 4.27–40.43), and scarred uterus (unadjusted OR 2.31, 95% CI 1.56–3.39 and adjusted OR 1.99, 95% CI 1.28–3.10). A significant difference was also observed for gestational hypertension, but we did not consider this factor, as discussed above. We also did not observe statistically significant associations for the other evaluated variables.

**Figure 3:**
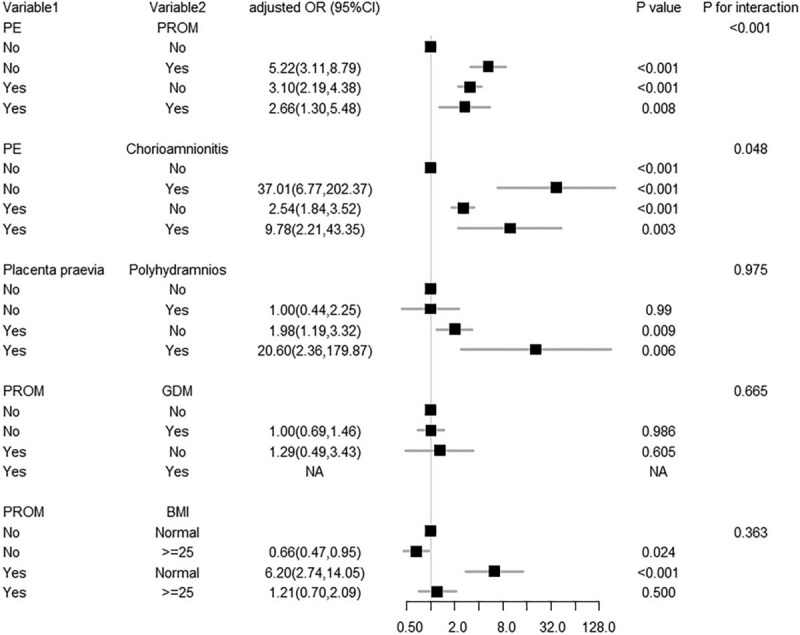
Association between 5 subgroups: PE and PROM, PE and chorioamnionitis, placenta and polyhydramnios, PROM and GDM, and PROM and BMI. Adjusted odds ratios of clinical characteristics and other diseases in Table 1 for preterm birth in different subgroups. BMI = body mass index, GDM = gestational diabetes mellitus, PE = preeclampsia, PROM = premature rupture of the membranes.

Notably, many pregnant women in our study exhibited more than 1 type of gestational complication. Therefore, we divided the 1607 women into different subgroups according to Table 1 and explored the relationships among the 6 independent factors identified in different subgroups. We found that 5 combinations of conditions were statistically significant in terms of heterogeneity (*P* < .05) (Tables 2–7). In order to study whether independent risk factors were more likely to be associated with preterm birth when combined with other diseases, we also conducted an interactive analysis of preterm birth in these 5 groups of diseases (Fig. 3).

**Table 2:**
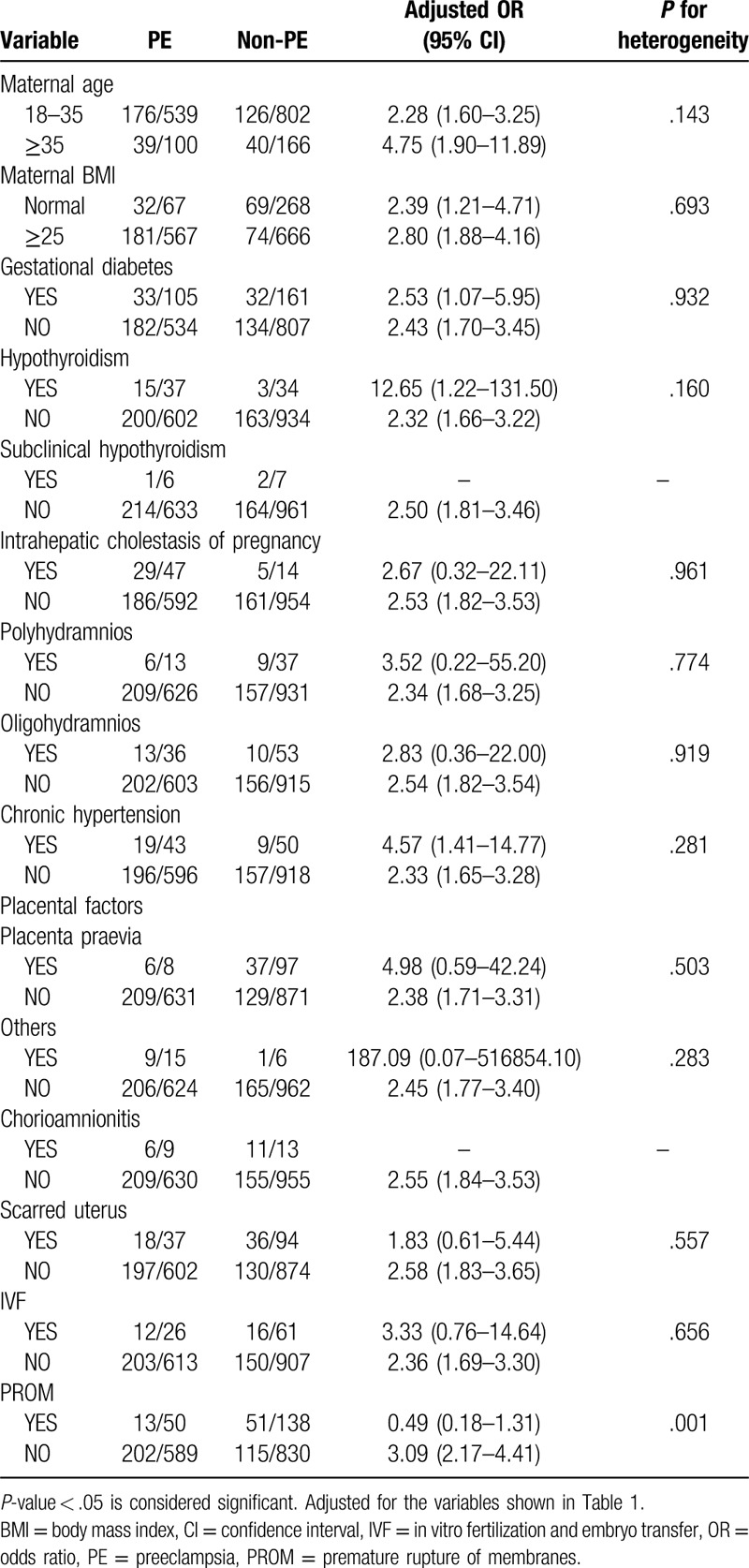
Heterogeneity between preeclampsia (PE) and other factors.

**Table 3:**
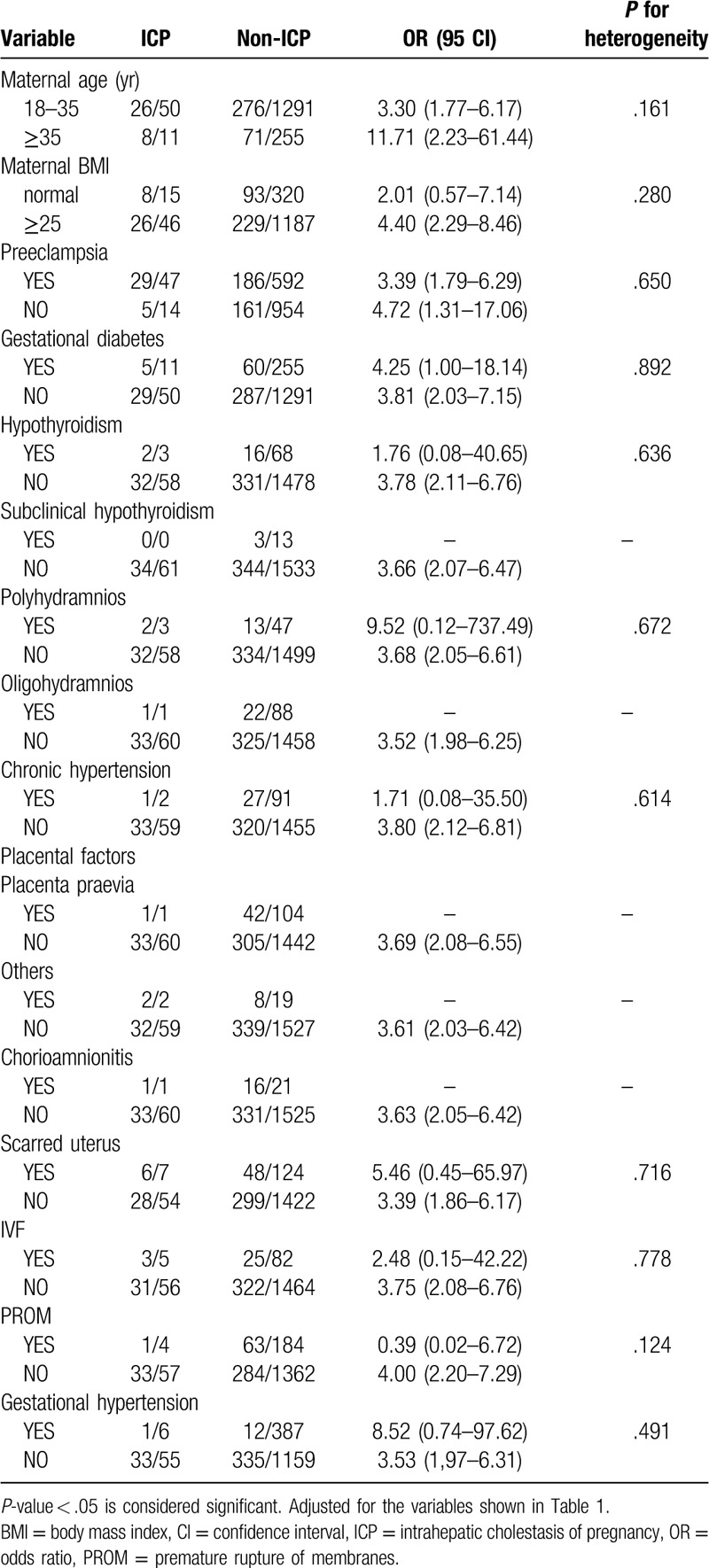
Heterogeneity between intrahepatic cholestasis of pregnancy (ICP) and other factors.

**Table 4:**
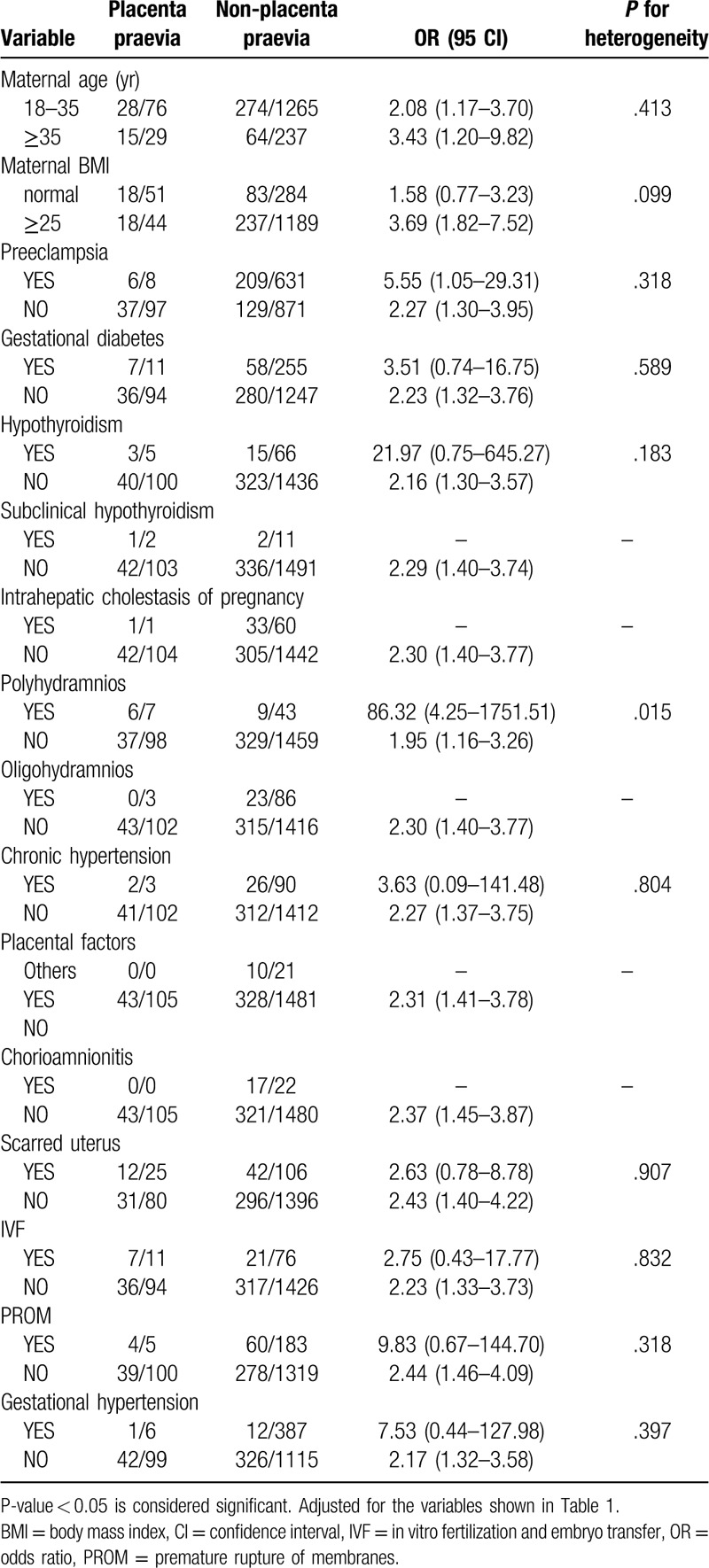
Heterogeneity between placenta praevia and other factors.

**Table 5:**
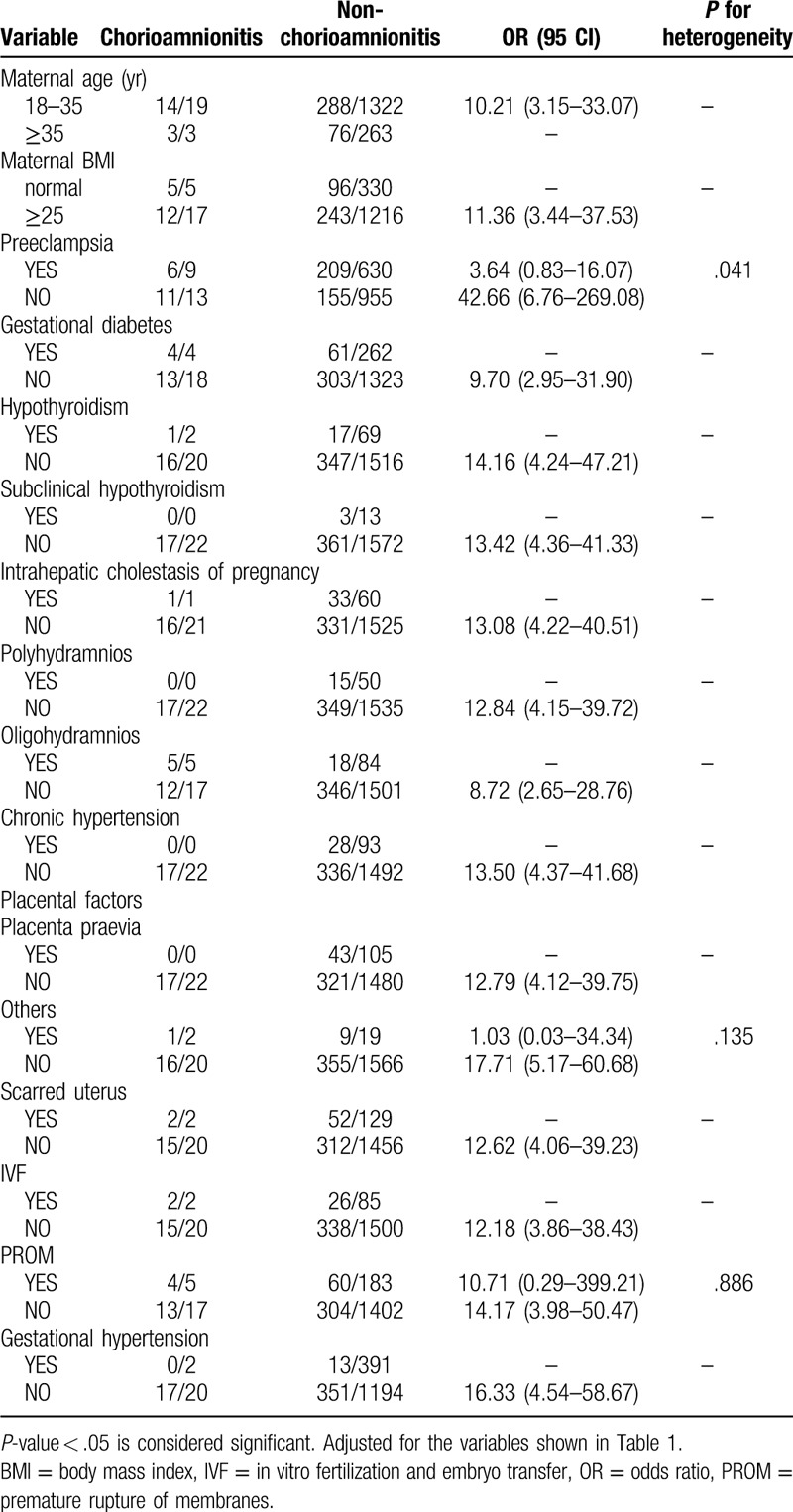
Heterogeneity between chorioamnionitis and other factors.

**Table 6:**
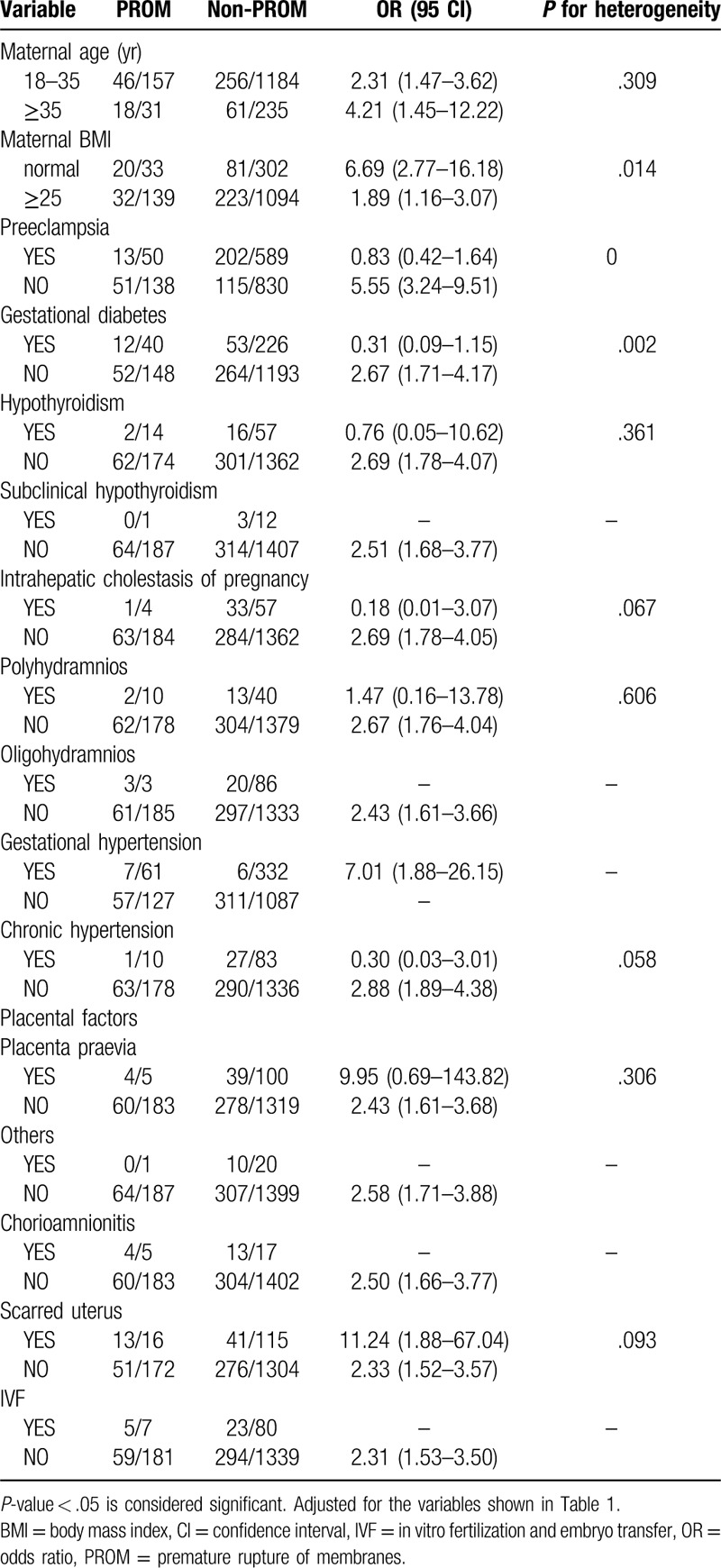
Heterogeneity between PROM and other factors.

**Table 7:**
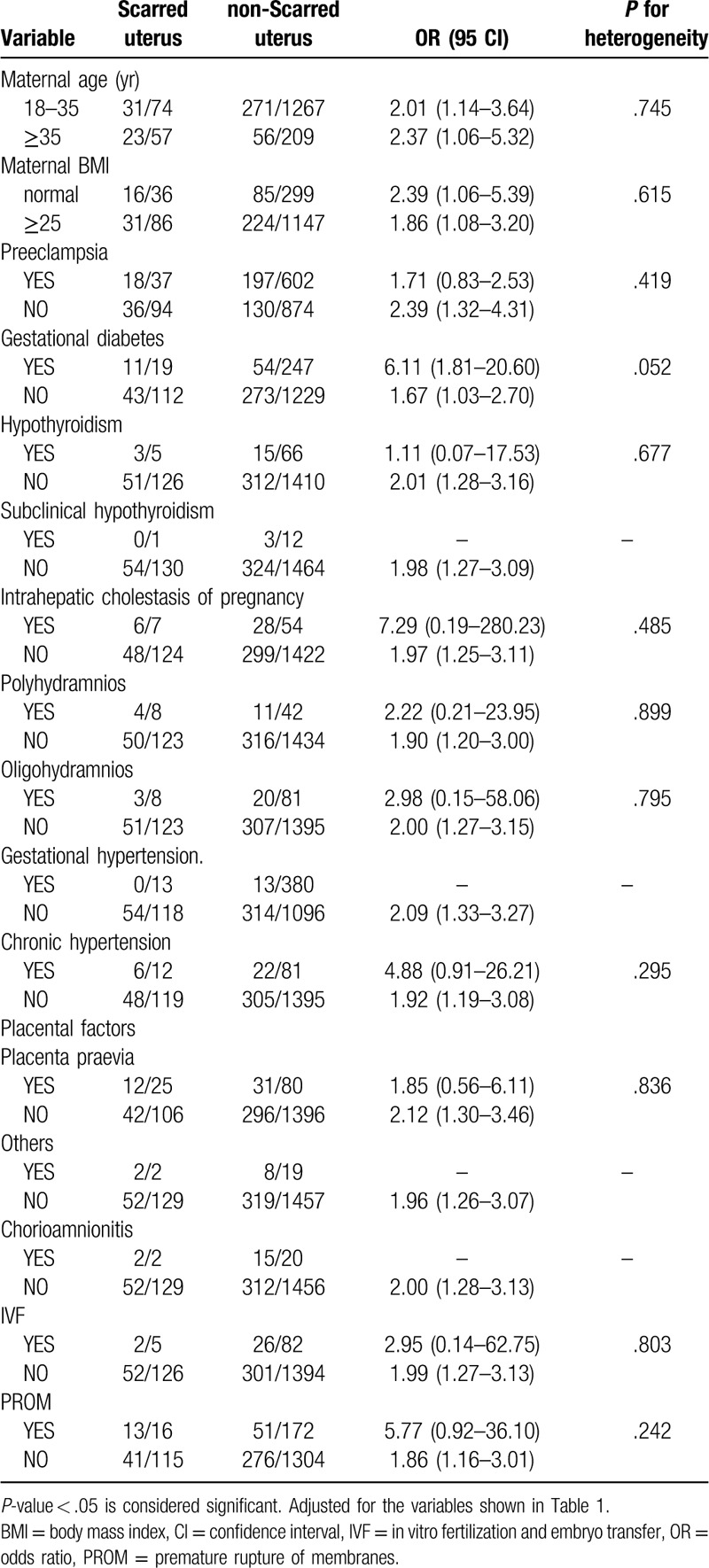
Heterogeneity between scarred uterus and other factors.

We found that women with PE and overlapping chorioamnionitis were more likely to have preterm births than women without chorioamnionitis (adjusted OR 9.78 vs 2.54, *P* for interaction = .048; Fig. 3). However, women with only chorioamnionitis had a higher risk of preterm birth than those with both diseases (adjusted OR 37.01 vs 9.78, *P* for interaction = .048; Fig. 3). Moreover, women with PE and overlapping PROM were more likely to undergo preterm birth than those who only had PE or PROM (adjusted OR 2.66 vs 3.10 vs 5.22, *P* for interaction < .001; Fig. 3), although PE and PROM are both independent risk factors.

Thus, there was heterogeneity for risk of preterm birth for women with placenta previa and polyhydramnios at the same time compared with women who had placenta previa without polyhydramnios (*P* for heterogeneity = .015; Table 4). Figure 3 shows that women who developed placenta previa with polyhydramnios had a higher risk of preterm birth than those who only developed one of the diseases, but the result is not statistically significant (*P* = .975; Fig. 3). Similarly, BMI had an effect on the risk of premature birth for women with PROM (*P* for heterogeneity = .014; Table 6), but there appears to be no interaction between these factors. Although we did not observe gestational diabetes mellitus (GDM) as an independent risk factor in our study (*P* = .954; Fig. 2), GDM appeared to be associated with the risk of premature birth for women with PROM (*P* for heterogeneity = .002; Table 6), but we observed no interaction between these factors.

## 4 Discussion

In this study involving women with singleton pregnancies, we identified 6 factors independently associated with preterm birth. Our data showed that PE is the leading primary independent medical factor related to preterm birth (56.4%) among the evaluated factors, followed by PROM (16.8%), scarred uterus (14.2%), placenta previa (11.3%), ICP (8.9%), and chorioamnionitis (4.5%). These are common diseases that require further attention.

PE is a serious disease that only occurs in pregnancy after 20 weeks. Most of the women who develop PE may need to undergo iatrogenic delivery before 37 weeks gestation.^[8]^ Additionally, the underlying disease process may increase the risk of spontaneous preterm birth.^[9,10]^ This idea is consistent with our findings. In our study, as an independent risk factor, we found that the odds of preterm birth among women with PE were 2.46 times greater (95% CI 1.78–3.40) than that of women without the condition. Chorioamnionitis, characterized by inflammation of embryonic membranes, was also an independent risk factor for preterm birth. Previous studies have reported that preterm birth is the result of chorioamnionitis, and approximately 25% of preterm births can be attributed to chorioamnionitis.^[11]^ In our study, the odds of preterm birth among women with chorioamnionitis were 13.14 times higher (95% CI 4.27–40.43) than those of women without chorioamnionitis. We also found that women with PE and overlapping chorioamnionitis had a higher risk of preterm birth than women only with PE.

The production of proinflammatory mediators is an important factor associated with preterm birth and infection.^[12]^ The effects of pregnancy complications on the placental microbiota are still being explored, but to some extent, the results of the current study may indicate that the coexistence of PE and chorioamnionitis can impact preterm birth (Fig. 3). Therefore, we need give high priority to the treatment of pregnant women with PE combined with chorioamnionitis. Clinically, the diagnosis of chorioamnionitis includes fever, uterine tenderness, maternal or fetal tachycardia, maternal leukocytosis, and malodorous uterine discharge. In our study, women with PE and chorioamnionitis had no higher risk of preterm birth than women with only chorioamnionitis (Fig. 3). As shown in Table 2, only 13 of the 1607 women developed chorioamnionitis without PE, but 11 of these women had preterm births, which may explain this result.

In addition, the odds of preterm birth among women with PROM were 2.5 times greater (95% CI 1.68–3.77) than that of women without this condition in our study. PROM is always associated with inflammation and infection, which commonly cause spontaneous preterm birth.^[13,14]^ We performed an interactive analysis of the 2 risk factors and found that PE and PROM interactively impact preterm birth (*P* < .001). Interestingly, compared to women with both PE and PROM, women who developed PE or PROM were more likely to deliver before 37 weeks. A previous study reported that the risk for preterm birth was 7.72 times higher for women with iatrogenic deliveries and PE than for women with spontaneous deliveries and no PE.^[15]^ Some unknown mechanisms may affect each other and lead to this result. To our knowledge, similar conclusions have not been found in other reports on the subject, as other articles are typically focused on the study of independent risk factors without interactions. A larger sample size may be required to verify the validity of this conclusion.

Krupa et al reported that vaginal bleeding caused by placenta previa is associated with a high risk of preterm birth.^[16]^ In our study, we found that placenta previa is a significant independent risk factor for preterm birth (adjusted OR 2.29, 95% CI 1.40–3.75, *P* < .001). Additionally, heterogeneity exists for preterm birth between women who develop placenta previa with polyhydramnios and those without polyhydramnios. However, we observed no interaction risk for preterm birth between placenta previa and polyhydramnios. Therefore, we must adequately screen pregnant women for both diseases. The risk of preterm birth appears to be approximately 20 times greater for women who develop placenta previa and polyhydramnios than for women who have only one of these diseases (Fig. 3), although this was not statistically significant in our sample. It may be necessary to obtain a larger sample size to obtain a more precise estimate and 95% CIs.

Our study shows that PROM complicates 8% to 10% of all pregnancies and is a significant independent risk factor for preterm birth (adjusted OR 2.52, 95% CI 1.68–3.77, *P* < .001), although some articles have reported that 60% of PROM occurs at term.^[17,18]^ GDM is a common gestational complication of women, and a previous study reported that GDM complicates 1% to 14% of pregnancies in the United States.^[19]^ However, in our study, we saw no interaction risk for preterm birth between PROM and GDM, despite heterogeneity in the subgroup. Notably, women with PROM and normal BMI were more at risk for preterm birth than women with a BMI ≥25 (adjusted OR 6.20, 95% CI 2.74–14.05 vs adjusted OR 1.21, 95% CI 0.70–2.09). This result may be influenced by the population base, because in the present study, there were many women with BMI ≥25 (n = 1233). Chorioamnionitis is usually caused by a bacterial infection in the presence of a ruptured membrane, and is considered a contributing factor to preterm birth.^[10]^ There were only 5 women who developed PROM with chorioamnionitis, and 4 of these women had preterm births. Thus, because of the low numbers, we could not perform statistical calculations. The presence of a scarred uterus seems to have no relationship with other diseases with regard to preterm birth, despite being an independent risk factor.

## 5 Limitations

Although we studied many variables that are often related to preterm birth, the list of risk factors is quite long. We only studied a few of them, and our study was single-center and retrospective in nature. We could not consider other important potential risk factors, such as working long hours or performing hard physical labor under stress, which may also be associated with an increase in preterm birth,^[20]^ because these factors were not reported in the medical records. Additionally, due to the sample size and imprecise estimates, we may have missed some significant associations, and it may be necessary to conduct multi-center research for the follow-up study.

## 6 Conclusion

Approximately 15 million babies are born prematurely every year in the world (more than 1 in 10), and this number seems to be increasing. Every year, more than one million deaths are estimated to result from the associated complications.^[4]^ Describing risk factors associated with preterm birth will be very useful for identifying high-risk pregnancies, and interventions for these risk factors may be important for preventing preterm birth. Similarly, the interaction between diseases involved in preterm birth cannot be ignored, as we observed for PE and chorioamnionitis in our study. Perhaps we can find more optimal methods to prevent preterm birth, such as studying the interaction between the pathogenesis of multiple diseases, or developing more targeted interventions for women who experience these, and who may be at higher risk of preterm birth.

## Author contributions

**Methodology:** Yating Qian.

**Resources:** Ruizhe Jia.

**Software:** Mingming Gao, Lei Zhang.

**Supervision:** Hongjuan Ding.

**Writing – original draft:** Jin Huang.

**Writing – review & editing:** Jin Huang.
